# Social inequalities in mental disorders and substance misuse in young adults: A birth cohort study in Southern Brazil

**DOI:** 10.1007/s00127-018-1526-x

**Published:** 2018-05-02

**Authors:** Fernando C. Barros, Alicia Matijasevich, Iná S. Santos, Bernardo L. Horta, Bruna Gonçalves C. da Silva, Tiago N. Munhoz, Seena Fazel, Alan Stein, Rebecca M. Pearson, Luciana Anselmi, Luis Augusto Rohde

**Affiliations:** 1 Post-Graduate Course in Health and Behavior, Catholic University of Pelotas, Rua Mal. Deodoro, 1160, 96020-220 Pelotas, RS Brazil; 2 Department of Preventive Medicine, Faculty of Medicine, University of São Paulo, São Paulo, Brazil; 3 Postgraduate Program in Epidemiology, Federal University of Pelotas, Pelotas, Brazil; 4 Department of Psychiatry, University of Oxford, Oxford, UK; 5 School of Social and Community Medicine, University of Bristol, Bristol, UK; 6 Department of Psychiatry, Hospital de Clínicas de Porto Alegre, Federal University of Rio Grande do Sul, National Institute of Developmental Psychiatry for Children and Adolescents, Porto Alegre, Brazil

**Keywords:** Mental disorders, Substance misuse disorders, Young adulthood, Alcohol abuse, Socioeconomic status, Life course epidemiology, Social determinants of health

## Abstract

**Purpose:**

To investigate the association between mental disorders and substance misuse at 30 years of age with gender, socioeconomic position at birth, and family income trajectories.

**Methods:**

The 1982 Pelotas Birth Cohort was used; all 5914 children born alive at hospital were originally enrolled (99.2% of all city births). In 2012, 3701 subjects were located and interviewed (68% retention rate). Mental disorders and substance misuse were assessed, and their prevalence analysed according to gender, socioeconomic status at birth, and four different income trajectories: always poor, never poor, poor at birth/non-poor at age 30, and non-poor at birth/poor at age 30.

**Results:**

While women presented higher prevalence of mental disorders, substance misuse was much more frequent among men. Individuals in the lowest income quintile at birth presented 2–5 times more mental disorders and substance misuse than those in the highest quintile. Young adults who were always poor or were not poor at birth but were poor at 30 years of age had a higher prevalence of mental disorders than the other groups.

**Conclusions:**

The high rates of mental disorders and lifetime suicide attempts in young adults, especially those who were always poor or became poor after childhood, suggest that recent socioeconomic-related stressful situations may have a higher impact on the current mental health than events earlier in life. However, we could not identify at what specific ages socioeconomic changes were more important.

**Electronic supplementary material:**

The online version of this article (10.1007/s00127-018-1526-x) contains supplementary material, which is available to authorized users.

## Introduction

Mental and substance use disorders are highly prevalent worldwide. The WHO World Mental Health Survey conducted in 28 countries found the 25th–75th percentile inter-quartile range prevalence estimates of mental health disorder (combining anxiety, mood, externalizing, and substance use disorders) to be in-between 18–36% [1]. Recent recalculations of the global burden of mental illness suggested that it accounts for 32% of years lived with disability (YLDs) and 13% of disability-adjusted life-years (DALYs), instead of the earlier estimates suggesting 21% of YLDs and 7% of DALYs [2].

Mental disorders have different aetiologies, are more common in women, and particularly affect individuals with accumulating social and family disadvantage, living mostly in low–middle-income countries (LMICs) [3]. In a systematic review of the literature reporting findings from these countries, more than 70% of the 115 reviewed papers showed an association between different measures of poverty and common mental disorders [4].

Although several investigations have been carried out in LMICs, the so-called 10/90 gap (only 10% of mental health research is conducted in LMICs where almost 90% of population live) still remains [5, 6]. Methodological limitations often found in LMICs investigations make it difficult to determine if social determinants are really important for mental disorders or if they are just markers for something else. For instance, two key areas where much debate remains are measurement of socioeconomic status (SES) and directionality of effects [4, 7]. Finally, disentangling effects of social inequality from poverty is particularly difficult in LMICs [6].

In this context, more information is needed on the relationship between SES and mental and substance use disorders in LMICs. It remains to be determined, for example, if social deprivation affects different categories of psychiatric disorders to the same extent. In addition, as cross-sectional designs cannot clearly indicate the directions of the association between poverty and mental disorders, longitudinal studies are required to define in which developmental periods social deprivation is more likely to affect mental health and how lifetime changes in SES affect these outcomes. In this regard, a systematic review on the association between SES in the early life and drug use in young adults relying only on longitudinal studies showed consistent evidence to support an association between lower childhood SES and later drug use, primarily cannabis use [8]. Furthermore, in a Swedish study, trajectories of childhood family income over a 12-year period were assessed in over 500,000 individuals [9]. Among the five identified income trajectories, the constant low and the downward trajectories were particularly associated with later mental disorders.

One important question is whether possible associations of early family deprivation and adult mental health may be changed by subsequent family socioeconomic improvement. In a natural experiment in the United States, children whose families increased their income due to external reasons showed reduced symptoms of conduct and oppositional disorders, although anxiety and depression were not affected [10].

The aims of the current study were to estimate the prevalence of mental disorders and alcohol misuse and illegal substance use at 30 years of age, in a population-based cohort followed since birth in Southern Brazil. These outcomes were analysed according to gender, SES status at birth, and family income trajectories constructed with measures obtained at several time points.

## Methodology

During 1982, all maternity hospitals in Pelotas, Southern Brazil, were visited daily and all 5914 babies whose families lived in the urban area of the city were approached and their mothers interviewed soon after delivery. These births accounted for 99.2% of all births occurring in the city in that year. This cohort was followed in several occasions [11, 12].

Initial follow-up visits were conducted in 1984 and 1986. In 1984, 4934 children of the cohort were traced (87%), and in 1986, we were able to find 4934 (84%) of the original participants. In 1997, when the children were aged 14–15 years, we visited a systematic sample of 27% of the city’s census tracts, and were able to find 1,076 children, corresponding to 72% of the target population. In 2000, at age 18 years, during the compulsory Army recruitment examination, we obtained consent to interview and examine 2250 of the male members of the cohort, corresponding to 80% of the men in the original cohort. In the next year, 2001, we revisited all households in the 70 census tracts that had been visited in 1997, and conducted an extensive interview with all women and a shorter one with men, who had already been examined the preceding year. In this 2001 visit, we were able to reach 1,589 subjects, corresponding to 70% of our target. In 2004, a new visit was made to all cohort members when they were aged 22 years, and 4297 subjects were located, corresponding to 77.4% of the original cohort. Finally, from June 2012 to February 2013, the cohort members were invited to visit our research clinic, to be interviewed and examined. A total of 3701 subjects were interviewed, and 3542 completed the psychological assessment (retention rate = 68.1% of all those known to be alive − 325 are known to have died).

Interviews during childhood were conducted only with the mother or with the person with guardianship of the child. During adolescence, both parents/guardians and adolescents were interviewed. The 2012 interview was conducted with the young adult.

All phases of the study were approved by the Ethical Committee of the Federal University of Pelotas, Brazil.

### Analysis of family income trajectories

Family income data were obtained in 1982 (birth, *n* = 5914), 1984 (mean age 2 years, *n* = 4961), 1986 (4 years, *n* = 4737), 1997 (15 years, *n* = 1075), 2001 (19 years, *n* = 1589), 2004 (22 years, *n* = 4296), and 2012 (30 years, *n* = 3483). In the perinatal visit, as we had five fixed groups of income which were unequal in size, we conducted a principal component analysis with four linked socioeconomic variables—delivery payment mode, mother’s schooling, height, and skin color. The first component was used to derive a score that was then used to rank individuals within family income groups. Cut-off points were then found within each category, so that three or five nearly equal sized groups were formed. For all other visits continuous values of total family income were obtained, which were then recoded into groups of equal size. From 2001 onwards, the family income also included the subjects own earnings, and, especially in the 30 year visit, if they no longer lived with their parents, the earnings were those of their own families, and not those from their parents.

Longitudinal trajectories of family income were studied using a semiparametric, group-based modelling approach [13, 14]. Group-based trajectory modelling is a particular form of finite mixture modelling, designed to identify rather than assume groups or clusters of individuals following similar developmental trajectories. A polynomial function was used to model the relationship between an attribute (i.e., monthly family income) and age or time [13–15]. The models were estimated with the Stata procedure “traj”[16]. Valid data from at least three time points are required to estimate group-based trajectories. We included 3498 subjects who satisfied this criterion and had information on mental health at age 30. The choice of number and shape of trajectories were based not only on the best fit of the model (maximum Bayesian information criteria, BIC), but also on the interpretability of the trajectories obtained [14]. We modelled trajectories of family income with information collected at seven time points: at birth, 2, 4, 15, 19, 22, and 30 years of age. Analyses were conducted specifying three-, four-, and five -group models. BIC improved in the four-group model and it emerged as the best fitting and most parsimonious model (− 10514.77, − 10492.71, − 10510.72 for the three-, four- and five-group models, respectively). Inspection of parameter estimates for the four-group model revealed that the constant term differed from zero for all four groups (Supplemental data file—Table S1). Two trajectories were best represented by a cubic term and the other two trajectories by quadratic term (Supplemental data file Figure S1). The average posterior probability (APP) of membership was 0.81, 0.60, 0.67, and 0.74 for group 1–group 4, respectively.

The first group, comprising 49.6% of the subjects, was represented by those individuals whose families always had a very small probability of belonging to the poorest first tertile of income (thereafter called, for the sake of simplification, “never poor”); group 2, with 15.9% of the cases, represented the individuals with high probability of being in the lowest tertile in the first four years of life, but a low probability of being in this poorest group between 20 and 30 years of age (“poor at birth, non-poor at age 30”); group 3 (13.9%) presented the opposite pattern: a small probability of being in the lowest tertile in the first 4 years of life, and then a high probability between 20 and 30 years of age (“non-poor at birth, poor at age 30”); finally, group 4 (20.6% of the subjects) comprised subjects who always had a high probability of belonging in the lowest income tertile (“always poor”).

### Analysis of mental disorders and use of substances at age 30

Trained psychologists performed the entire diagnostic evaluation and were supervised by an experienced senior psychologist. A general psychiatric assessment was performed with the Mini International Neuropsychiatric Interview (MINI) V5.0 [17], a short semi-structured diagnostic interview for DSM-IV and ICD-10 psychiatric disorders that provided prevalence estimates of current prevalence of common mental disorders. Because of logistic issues (i.e., the psychiatric interview was part of a larger follow-up assessment), only some MINI sections were performed. Modified modules for general anxiety disorder (GAD), social phobia, major depressive disorder (MDD), bipolar disorder (BD), and attention-deficit/hyperactivity disorder (ADHD) were applied. The MINI has been previously validated in Portuguese [18]. In primary health care in Brazil, the MINI exhibited κ values of 0.65–0.85, a sensitivity of 0.75–0.92, and a specificity of 0.90–0.99 when using diagnoses obtained by a psychiatrist [19].

Diagnoses were confirmed if individuals reported that their symptoms impaired their work, study, leisure, and their relationships with family and friends.

Self-administered computer questions on lifetime suicide attempt and substance use in the last month (cannabis, cocaine, and crack) were enquired confidentially.

Alcohol-use disorders were evaluated with AUDIT [20]; a cut-off point of 12 was adopted as this has shown the best sensitivity (0.84) and specificity (0.86) in a representative sample of a Pelotas, Brazil, adult population [21].

The Self-Report Questionnaire (SRQ-20), a screening instrument to assess the presence of depression and anxiety [22], was also used in 2012. The test consists of 20 questions about physical and psychological symptoms during the 30 days prior to the interview. The cut-off point considered as indicative of depression and/or anxiety disorder was six or more positive answers for women and eight or more positive answers for men, and the test presented a good sensitivity (83%) and specificity (80%) for indicating disorder against a psychiatric interview [23].

The SRQ-20 was also used in the previous follow-up visits to cohort members in 2000, 2001, and 2004. It was completed by 2236 men examined in 2000, during the Army recruitment; in the next year (2001), the test was used in the 920 women examined, out of the 1589 visits, as men had already being tested the previous year. In 2004, 4285 subjects answered the SRQ-20 questions.

Prevalence rates and 95% confidence intervals were obtained in the analyses of the association between mental health and substance use outcomes and exposures. Chi-square tests were employed in the statistical analyses, while Spearman correlation coefficients were utilized in the analyses of associations between ordinal variables.

G-computation [24] was used to estimate the direct effect of socioeconomic trajectory on depression and anxiety at 30 years as well as the indirect effect that was mediated through mental health at 22–23 years. In this model, maternal schooling was considered as a base confounder, and the participant schooling at 23 years as a post-confounder in the mediation analyses.

## Results

As this paper uses relative measures of SES—quintiles or tertiles of family income - the first table aims to contextualize the situation in which parents and children lived during the first 2 years of life. Table 1 disaggregates into quintiles of family income measured at birth some maternal reproductive results obtained in the perinatal interview, and parents education, family housing, and sanitation characteristics, obtained in the 1984 follow-up visit. At this time, information was also obtained on child morbidity and nutritional status. Regarding reproductive data, mothers in the lowest quintiles were more often teenagers, single, multipara, and smokers. Maternal and paternal educational levels were also markedly different, with the better-off quintile presenting nearly three times more years of schooling, compared to the poorest quintile. Water, sanitation, and ownership of refrigerator were also unequally distributed: nearly half of the households in the lowest quintile did not have these facilities. Deprivation was considered when the family lacked at least three of the following: piped water, sewage, refrigerator, and father or mother minimum level of education. Half the families in the lowest quintile, and 35% in the second lowest, were considered deprived, in comparison to 1.5% in the highest quintile group.

**Table 1:** Sociodemographic characteristics, morbidity history, and housing conditions of families belonging to different socioeconomic quintiles. 1982 Pelotas Birth Cohort (all values are percentages)

Variables	Socioeconomic quintiles					*P* value
	Lowest	2	3	4	Highest	
1982 (*N*)	1183	1178	1180	1185	1188	
Maternal age < 20	26.1 (23.6–28.6)	15.4 (13.3–17.4)	18.9 (16.7–21.1)	11.1 (9.3–12.9)	5.6 (4.3–7.0)	< 0.001
Maternal age 35+	8.0 (6.5–9.6)	12.5 (10.6–14.4)	7.2 (5.7–8.7)	9.7 (8.0–11.4)	12.2 (10.3–14.1)	0.04
Single mother	19.6 (17.3–21.9)	6.9 (5.4–8.3)	5.3 (4.1–6.6)	5.7 (4.4–7.1)	3.5 (2.4–4.5)	< 0.001
Smoking in pregnancy	43.9 (41.0–46.7)	38.3 (35.5–41.1)	35.7 (32.9–38.4)	34.3 (31.6–37.0)	25.8 (23.3–28.2)	< 0.001
3 + previous children	25.4 (23.0–27.9)	23.9 (21.5–26.4)	13.6 (11.7–15.6)	11.2 (9.4–13.0)	7.5 (6.0–9.0)	< 0.001
1984 (*N*)	929	994	1027	1045	989	
Mother < 5 years of schooling	59.4 (56.3–62.6)	53.9 (50.8–57.0)	24.1 (21.5–26.8)	15.6 (13.4–17.8)	5.6 (4.1–7.0)	< 0.001
Father < 5 years of schooling	52.7 (49.3–56.1)	43.6 (40.4–46.8)	28.7 (25.9 − 31.6)	14.6 (12.4–16.8)	5.9 (4.4–7.4)	< 0.001
Father hospitalized alcohol problems	11.4 (9.0–13.8)	9.4 (7.4–11.3)	6.3 (4.6–7.9)	3.7 (2.4–5.0)	2.0 (1.0–2.9)	< 0.001
Father hospitalized psychiatry	8.0 (6.0–10.1)	3.1 (1.9–4.2)	3.4 (2.2–4.6)	2.5 (1.5–3.6)	1.5 (0.7–2.3)	< 0.001
Mother hospitalized psychiatry	6.9 (5.2–8.7)	6.4 (4.9–8.0)	5.1 (3.7–6.4)	5.0 (3.6–6.3)	1.8 (1.0–2.7)	< 0.001
Child hospitalized due to diarrhoea or pneumonia	29.7 (26.8–32.6)	22.0 (19.5–24.6)	18.6 (16.3–21.0)	13.4 (11.3–15.5)	5.5 (4.0–6.9)	< 0.001
Child stunting	26.6 (23.7–29.5)	19.4 (16.9–21.8)	8.5 (6.8–10.2)	5.6 (4.2–7.0)	2.7 (1.7–3.8)	0.001
No piped water inside the house	49.1 (45.9–52.3)	37.6 (34.6–40.6)	20.7 (18.2–23.2)	10.2 (8.4–12.1)	1.9 (1.1–2.8)	< 0.001
No flush toilet	45.0 (41.9–48.2)	30.3 (27.5–33.2)	20.2 (17.7–22.6)	8.8 (7.1–10.5)	1.9 (1.1–2.8)	< 0.001
No refrigerator	56.4 (53.2–59.5)	35.1 (32.1–38.1)	22.9 (20.3 − 25.5)	9.2 (7.4–10.9)	2.3 (1.4–3.3)	< 0.001
Family deprivation	51.5 (48.2–54.7)	35.0 (32.0–38.0)	16.3 (14.0–18.5)	5.9 (4.5–7.4)	1.5 (0.7 − 2.3)	< 0.001

Regarding stressful deprivation early in life, nearly one in three children from the poorest families has been hospitalized at least once due to diarrhoea or pneumonia in the first 2 years of life, this proportion being 5.5% for the better-off. In addition, the prevalence of severe chronic malnutrition at 2 years of life was 26.6% among the poorest group, being nearly ten times less in the richest group.

Table 2 presents the prevalence of the main categories of investigated mental disorders—severe depression, generalized anxiety, bipolar disorder, social phobia, and ADHD—and of lifetime suicide attempts. It also shows the prevalence of alcohol abuse, and use of cannabis, cocaine, and crack. With the exception of bipolar disorders and ADHD, all other mental disorders were nearly twice as frequent in women as in men. Generalized anxiety disorder affected 8% of the total population (11.6% of women), while 5.0% of the whole group presented with major depression (7% in women). We have also used a composite index of psychiatric morbidity, combining one or more of the diagnosed mental disorders. The prevalence was 19.2% for women and 9.4% for men. SRQ-20 test was positive in 21.3% of men and 27.1% of women.

**Table 2:** Prevalence (95%CI) of mental and substance use disorders at 30 years of age, by gender. 1982 Pelotas Birth Cohort Study (*n* = 3512) (all values are percentages)

Mental disorders	1982			*P* value
	Men (%)	Women (%)	All (%)	
*N*	1712	1816	3528	–
Major depressive disorder	3.0 (2.2–3.8)	7.0 (5.8–8.2)	5.0 (4.3–5.8)	0.001
Generalized anxiety disorder	4.3 (3.4–5.3)	11.6 (10.1–13.0)	8.0 (7.1–8.9)	0.001
Bipolar disorder	1.9 (1.2–2.5)	1.7 (1.1–2.3)	1.8 (1.4–2.2)	0.4
Social phobia	2.2 (1.3–2.6)	5.1 (4.1–6.1)	3.6 (3.0–4.2)	0.001
ADHD	2.0 (1.3–2.7)	2.7 (1.9–3.4)	2.4 (1.9–2.9)	0.1
Any of the investigated mental disorders*	9.4 (8.0–10.8)	19.2 (17.3–21.0)	14.4 (13.3–15.6)	0.001
SRQ-20 (positive)	21.3 (19.4–23.2)	27.1 (25.1–29.1)	24.3 (22.9–25.7)	< 0.001
Lifetime suicide attempt	4.7 (3.7–5.7)	8.3 (8.3–9.6)	6.6 (5.8–7.4)	0.001
Self-reported alcohol and substance use				
Alcohol abuse (audit ≥ 12)	18.0 (16.2–19.8)	3.9 (3.0–4.7)	10.7 (9.7–11.7)	0.001
Cannabis use last month	13.4 (11.8–15.0)	4.3 (3.4–5.2)	8.7 (7.7–9.6)	0.001
Cocaine use last month	9.9 (8.5–11.3)	2.4 (1.7–3.1)	6.0 (5.2–6.8)	0.001
Crack use last month	1.7 (1.1–2.4)	0.5 (0.2–0.8)	1.1 (0.7–1.4)	0.001

*One or more of the mental disorders: major depressive disorder, generalized anxiety disorder, bipolar disorder, social phobia, and ADHD

The opposite gender situation was found for misuse of alcohol and use of substances, when the prevalence among men was typically three to four times higher than that of women. Harmful use of alcohol was observed in one in every five men, a high prevalence of cannabis (13.4%) and cocaine (9.9%) use was also observed among men. Crack use was self-reported by 1.7% of men and 0.5% of women.

The 24.3% young adults who scored above the threshold for the SRQ-20 screen at 30 years of age had a much higher risk of exhibiting all mental disorders and alcohol misuse and substance use. The risk of depression or anxiety was 10 times higher, and the risk was 3–5 times higher for the other mental disorders and suicide attempt. For alcohol misuse and substance use, the increased risk in SRQ positive subjects was 3 times higher for crack use and 1.6 times higher for the other substances.

Strong associations were also observed between SRQ-20 tests performed at 18–19, 23, and 30 years of age, and mental disorders and alcohol abuse and substance use measured at age 30, although the magnitude of the risk was less pronounced. However, positive subjects in 2000–2001 and 2004 still presented a three times higher risk of depression or anxiety in 2012, this increased risk was also observed for other mental disorders and alcohol abuse and substance use. All the described differences were statistically significant (*P* < 0.05).

**Fig. 1:**
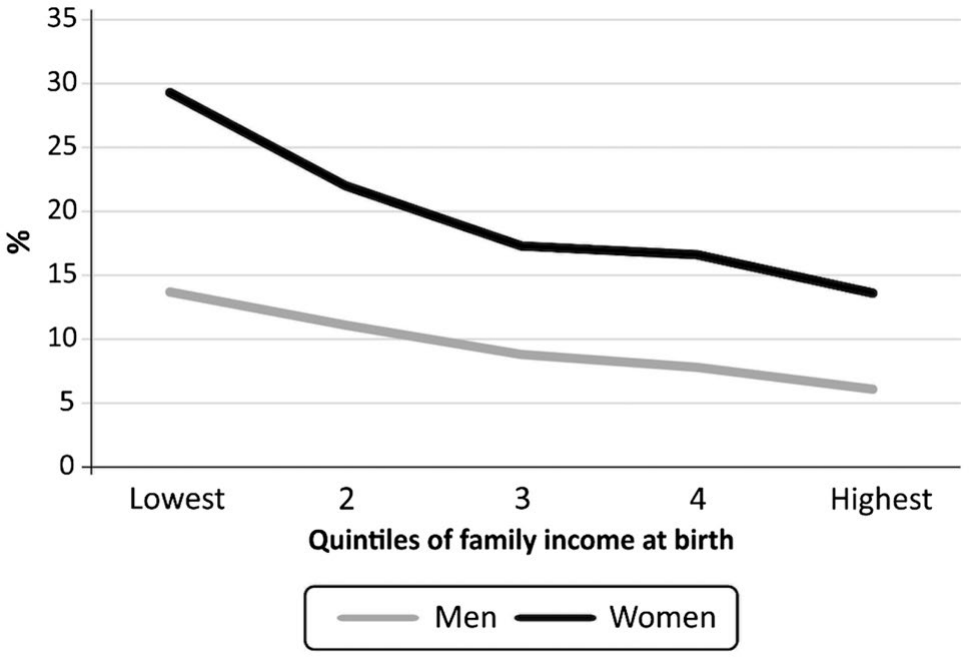
Prevalence of one or more mental disorders at 30 years of age by gender and quintiles of family income at birth. 1982 Pelotas Birth Cohort

Table 3 shows the prevalence of mental disorders and substance misuse at 30 years of age disaggregated by family income quintiles measured at birth. Mental disorders—with the exception of ADHD—and suicide attempt were 2–5 times more frequent in the poorest quintile relative to the highest. Regarding alcohol and substance use, cocaine and crack use were more frequent among the poor, while alcohol abuse and cannabis use showed no social gradients. Figure 1 depicts the prevalence of the composite of mental disorders by gender and SES. The prevalence among women was higher than among men in all socioeconomic groups, with women in the poorest quintile showing a 29.3% prevalence of mental disorders, compared to 6.1% among men in the highest quintile.

**Table 3:** Prevalence of mental and substance use disorders at age 30 years by socioeconomic quintiles at birth

Mental disorders	Socioeconomic quintiles					*P* value
	Lowest	2	3	4	Highest	
*N*	620	723	748	752	685	–
Major depressive disorder	6.9 (4.9–8.9)	6.1 (4.3–7.8)	4.8 (3.3–6.3)	4.1 (2.7–5.5)	3.5 (2.1–4.9)	< 0.001
Generalized anxiety disorder	12.4 (9.8–15.0)	7.8 (5.9–9.8)	6.7 (4.9–8.5)	7.5 (5.6–9.3)	6.4 (4.6–8.3)	< 0.001
Bipolar disorder	3.0 (1.7–4.3)	1.8 (0.8–2.7)	1.7 (0.8–2.6)	2.0 (1.0–3.0)	0.6 (0.01–1.1)	0.005
Social phobia	6.3 (4.4–8.3)	3.8 (2.4–5.2)	4.0 (2.6–5.4)	2.0 (1.0–3.0)	2.2 (1.1–3.3)	< 0.001
ADHD	2.9 (1.6–4.2)	1.8 (0.8–2.8)	2.0 (1.0–3.0)	2.1 (1.0–3.0)	3.1 (1.8–4.4)	0.432
Any mental disorder*	21.6 (18.3–24.9)	16.0 (13.3–18.7)	13.2 (10.7–15.6)	12.1 (9.8–14.5)	10.2 (7.9–12.5)	< 0.001
SRQ-20 (positive)	32.8 (29.2–36.4)	26.7 (23.5–29.9)	23.7 (20.7–26.7)	23.6 (20.6–26.6)	15.2 (12.5–17.9)	< 0.001
Lifetime suicide attempt	9.5 (7.2–11.8)	7.9 (6.0–9.9)	5.1 (3.6–6.7)	6.4 (4.7–8.2)	4.2 (2.7–5.7)	< 0.001
Self-reported alcohol and substance use						
Alcohol abuse (audit ≥ 12)	11.7 (9.2–14.2)	10.6 (8.4–12.9)	8.5 (6.5–10.5)	10.8 (8.6–13.0)	12.1 (9.6–14.5)	0.197
Cannabis use last month	9.8 (7.4–12.1)	7.2 (5.3–9.1)	6.5 (4.8–8.3)	9.2 (7.1–11.2)	10.9 (8.5–13.2)	0.185
Cocaine use last month	7.4 (5.3–9.4)	6.7 (4.8–8.5)	6.7 (4.9–8.5)	6.0 (4.3–7.7)	3.4 (2.0–4.7)	0.003
Crack use last month	1.8 (0.7–2.9)	1.6 (0.6–2.5)	0.8 (0.2–1.4)	1.3 (0.4–2.0)	0.1 (0.0–0.4)	0.004

1982 Pelotas Birth Cohort Study (*n* = 3310) (all values are percentages)

*One or more of the mental disorders: major depressive disorder, generalized anxiety disorder, bipolar disorder, social phobia, and ADHD

Alcohol misusers presented a 2.3 times higher risk of bipolar disease (*P* = 0.004), this risk being 1.8 times higher for ADHD (*P* = 0.044) and 1.5 times higher for depression (*P* = 0.033). In addition, they were much more likely to be engaged in substance use, such as cocaine (7.7 times increased risk), the risk being 5.0 times higher for crack and 4.5 times higher for marijuana use (all last *P* values < 0.0001).

Table 4 describes the prevalence of mental disorders, suicide attempt, and alcohol misuse, and substance use for the four groups of income trajectories. The most common observed pattern was that the group whose families were always poor presented the highest prevalence, and those who were never poor, the lowest. For the other two groups, those young adults whose families started their lives in a situation of poverty, but improved their socioeconomic situation afterwards (“poor at birth, non-poor at age 30”) tended to present with a lower prevalence of mental disorders than those who started their lives in a better-off situation, but became poor at 30 years of age. When only these two intermediate groups were compared, the prevalence of generalized anxiety disorder was significantly higher in the non-poor, then poor group (11.9%) than in the poor, then non-poor young adults (7.9%); *P* = 0.037. For all other conditions, the differences between the two intermediate groups were not statistically significant.

**Table 4:** Prevalence of mental and substance use disorders according to family socioeconomic trajectories between birth and 30 years of age

Mental disorders	Socioeconomic trajectories 1982–2012				*P* value
	Always poor	Non-poor, then poor	Poor, then non-poor	Never poor	
*N*	722	555	487	1734	–
Major depressive disorder	7.8 (5.8–9.7)	7.2 (5.1–9.4)	5.3 (3.3–7.3)	3.1 (2.3–3.9)	< 0.001
Generalized anxiety disorder	11.7 (9.4–14.1)	11.9 (9.2–14.6)	7.9 (5.5–10.3)	5.3 (4.3–6.4)	< 0.001
Bipolar disorder	2.4 1.2–3.5)	3.1 (1.6–4.5)	1.8 (0.7–3.0)	0.9 (0.4–1.3)	0.001
Social phobia	6.5 4.7–8.3)	3.4 (1.9–4.9)	2.5 (1.1–3.9)	2.5 (1.8–3.2)	< 0.001
ADHD	3.8 (2.4–5.2)	2.0 (0.8–3.2)	0.6 (0.0–1.3)	2.4 (1.7–3.2)	0.005
Any mental disorder*	22.4 (19.4–25.5)	19.0 (15.7–22.3)	12.4 (9.4–15.4)	9.6 (8.2–11.0)	< 0.001
SRQ-20 (positive)	34.7 (31.2–38.2)	30.2 (26.4–34.0)	21.0 (17.4–24.6)	17.3 (15.5–19.1)	< 0.001
Lifetime suicide attempt	10.6 (8.3–12.8)	9.0 (6.6–11.4)	6.4 (4.2–8.5)	3.6 (2.7–4.5)	< 0.001
Self-reported alcohol and substance use					
Alcohol abuse (audit ≥ 12)	10.5 (8.3–12.8)	10.8 (8.3–13.4)	10.9 (8.1–13.7)	10.6 (9.1–12.1)	0.995
Cannabis use last month	8.7 (6.6–10.8)	8.1 (5.7–10.4)	7.2 (4.9–9.6)	9.1 (7.8–10.5)	0.581
Cocaine use last month	7.6 (5.6–9.6)	7.2 (5.0–9.4)	6.9 (4.6–9.1)	4.6 (3.6–5.6)	0.015
Crack use last month	2.1 (1.0–3.1)	1.9 (0.7–3.1)	1.3 (0.3–2.3)	0.4 (0.1–0.6)	< 0.001

1982 Pelotas Birth Cohort Study (*n* = 3498) (all values are percentages)

*One or more of the mental disorders: major depressive disorder, generalized anxiety disorder, bipolar disorder, social phobia, ADHD

In relation to the time trajectory of the two intermediate groups from poverty to non-poverty, and vice versa, between birth and age 30, in the group that was poor at birth and then became non-poor at age 30, 85% of the families were still poor when the children were 4 years old, 20% were poor when they were aged 19, and only 4% were poor at age 22. On the other hand, in the non-poor at birth and poor at age 30 group, 19% were already poor at age 4, 50% were poor at age 18, and 72% were poor at age 22.

The prevalence at 30 years of age of any mental disorder in the poor, then non-poor group was estimated, separating those who were still poor at age 4, against those who were already non-poor at that age. The prevalence rates were 8.6 and 4.5%, respectively, and the *P* value was 0.494. The prevalence of any mental disorder at age 30 was also analysed in the non-poor, then poor group, dividing those who were already poor at age 4, versus those who were not. Prevalence rates were 17.0 and 19.4%, *P* value = 0.662.

We also explored the possibility that prior mental disorders might mediate effects of socioeconomic trajectories on mental disorders at 30 years of age. For these analyses, we used SRQ scores at 22 years of age (assessed in 2004) and depression or anxiety at age 30 as outcomes. We restricted our outcome to these two disorders, since SRQ-20 performance as a screening instrument is much better for anxiety or depression. The indirect effect was − 0.00808 (− 0.256, 0.0094), and there was not statistically significant evidence of interaction between exposure and mediator (*P* = 0.367). The indirect path explained 18% of the association between the exposure and the outcome.

## Discussion

This study investigates a birth cohort of 5,914 individuals for mental disorders and substance misuse. One key strength of this study is its longitudinal design, which permitted a follow-up over 30 years including nearly 70% of the original birth cohort in an urban area of one middle-income country. Follow-up rates were slightly higher among females, those who were born preterm, and those in the intermediate socioeconomic categories, whereas birthweight was not related to attrition [12].

Trained interviewers, using standardized instruments and diagnostic criteria, assessed mental disorders and alcohol and substance use at age 30. Measures of SES were obtained at seven different moments over the 30 yeaR lifespan. In addition, a screening instrument for mental disorders (SRQ-20) was used when the cohort members were aged 18, 23, and 30 years, and showed a high correlation in the three time periods, suggesting that, in many cases, current mental disorders were already present in late adolescence.

An important finding was the high prevalence of mental disorders and substance misuse in a young adult population in Southern Brazil, with considerably higher rates of mental disorders among women, while men presented a higher prevalence of alcohol and substance abuse. National surveys in USA have also reported higher prevalence of anxiety and mood disorders among women, while the pattern is for higher prevalence of impulsive-control disorder and substance use among men [25]. Interestingly, Moffitt et al. found similar patterns for internalizing disorders in the 1972–1973 Dunedin birth cohort at age 32 [26]. A higher prevalence of internalizing disorders among women has also been reported in ten European countries [27, 28].

We also found a high prevalence of self-reported lifetime suicidal attempts − 4.7% in men and 8.3% among women, reaching 10% among women in the two lowest income quintiles. However, a study of mortality due to external causes in Pelotas, 1996–2013, found that suicides were more common in men [29]. Thus, it appears that women are more likely to attempt suicide, while men are more likely to be successful in their attempts. In contrast, two meta-analyses on the Chinese general population have shown that the pooled prevalence of suicide attempts was 2.9% among adolescents [30] and 0.8% for a general population [31], being higher for women in both reviews. Gender differences in suicide attempts were also described in the World Mental Health Survey Initiative in Europe where any lifetime suicide attempt was found in 0.8–5.4% of women and 0.3–2.4% of men [27]. Recent evidence suggests that the 12-month prevalence rates of youth self-harm in low- and middle-income countries (LMICs) are similar to high-income countries [32]. Despite this evidence, the World Health Organization Atlas Project reported long-term systematic failure to allocate resources for mental health research, and policy and care services especially in LMICs [30, 33].

Another key finding of our study was that use of illegal substances, such as cannabis and cocaine is quite common, and that alcohol misuse is endemic among all socioeconomic groups, affecting nearly 20% of young men, which is confirmed by a Brazilian study of binge drinking, where the prevalence was even higher in high socioeconomic youngsters [34]. This is contrary to what is described in some European and North-American studies, where alcohol abuse appears to be more common in low socioeconomic young people [35, 36].

In addition, our data confirm the previous Brazilian reports of heavy episodic alcohol consumption in 16.5% of adults (≥ 18 years), according to eight large, nationally representative surveys [37]. The effects of alcohol use on mental health have been well established in the literature, and the acute use of alcohol increases the odds for suicide attempts, particularly in high doses [27, 38].

An important finding of this study was the large differences in prevalence of mental disorders and substance misuse observed among individuals belonging to different socioeconomic groups at the time of birth. This confirms the findings of a systematic review of the higher prevalence of mental disorders among poor young adults, when compared to those of higher socioeconomic level [39]. With the exception of ADHD, and alcohol and cannabis misuse, all the other investigated disorders were significantly more frequent among those persons whose families were more socioeconomically disadvantaged during their first years of life. As the measure of SES was taken prospectively from birth, this provides supports for social causation explanations of mental and substance use disorders. However, genetic and other early environmental confounding needs to be carefully examined to determine the extent to which these associations are independent.

Although the association of poverty with psychiatric disorders has long been known, [4], a contribution of this paper is that it measured socioeconomic changes over time, and then evaluated how these temporal changes could have affected mental health.

When comparing the four different socioeconomic trajectories between birth and age 30, we found that those individuals who had always lived in poverty presented the highest prevalence of mental disorders and substance misuse. Interestingly, comparing those who changed their family income trajectories between birth and early adulthood, the group of young adults who lived their first years of life in a poor environment, but whose families improved their economic conditions sometime after their childhood, showed a trend for a lower prevalence of mental disorders, compared to the group whose families were better-off in the first years of life, but then became poor in recent years.

Some of our findings suggest that recent socioeconomic-related stressful situations have a higher impact on the current mental health than events that occurred earlier in life. However, we could not pinpoint the time of change of different SES trajectories, which might have helped to understand when, in life, socioeconomic changes are particularly important.

As the current socioeconomic situation could have been affected by the previous mental disorders, we tested for mediation of mental health at 22 years of age, evaluated by the SRQ-20 in 2004, in the association between socioeconomic trajectories and depression or anxiety at age 30. Our analysis showed that the effect of mental health at age 22 could explain only 18% of the association. This indicates that, in this population, mental health assessed at age 22 does not mediate much of the association between SES trajectories and depression or anxiety at age 30. This finding suggests that reverse causation might be less important in explaining mental disorders at 30 years of age in our sample The effects of SES trajectories in mental health at 30 years of age do not seem to be just a by-product of the previous mental health symptoms.

In another study conducted in the same city in Southern Brazil with the 1993 Pelotas Birth Cohort at age 15, Anselmi et al. also found that those individuals who belonged to better-off families and later became impoverished presented higher prevalence of conduct problems than those from better-off families [40]. Furthermore, a recent large study in Sweden has also shown higher rates of mental disorders in individuals with a steady low or downward income trajectory [9].

The high prevalence of mental disorders and substance misuse in this young adult population, especially amongst its more impoverished members, is important from a public health and policy perspective. The situation is likely to worsen now as Brazil undergoes a harsh economic recession; it has been shown that economic crises that lead to job losses and income reductions, even in rich countries, have an important negative impact on mental health [41].

The striking socioeconomic differences in the prevalence of mental disorders and substance misuse revealed in this paper points to the urgent need of reducing social inequities. In fact, Rutter [42] has long maintained that, even if promoting better living standards do not necessarily lead to improvements in mental health, the key policy task is to reduce the widening disparity between the rich and the poor. In this regard, Brazil had been doing fairly well until recently, with a reduction in the Gini index from 0.64 in 1991 to 0.49 in 2012 [43], but this trend has been interrupted and a small increase has been observed in the last years.

Finally, our data demonstrate that new policies are urgently required to curb the epidemics of substance misuse, and in particular alcohol abuse. Among these policy initiatives, there is some consensus that increasing alcohol price seems to have a clear impact on consumption and damage [44, 45].

## Electronic supplementary material

Below is the link to the electronic supplementary material.

Supplementary material 1 (DOCX 16 KB)
