# Supplementary material for: Social inequalities in mental disorders and substance misuse in young adults: A birth cohort study in Southern Brazil
Source: Soc Psychiatry Psychiatr Epidemiol. 2018 May 2;53(7):717–26. doi: 10.1007/s00127-018-1526-x (PMC6003971; doi:10.1007/s00127-018-1526-x)
Supplement: Supplementary file 1 — Supplementary material 1 (DOCX 16 KB) [file 127_2018_1526_MOESM1_ESM.docx]

**Supplementary Table S1.** Descriptive and average posterior probabilities (APP) for the family income trajectories (n=3498).

| Trajectories of depressive symptoms | N (%) | APP (sd) | Parameters | Parameter Estimates | | |
| --- | --- | --- | --- | --- | --- | --- |
|  |  |  |  | B | (se) | p-value |
| 1 “Never poor” | 1734 (49.6) | 0.81 (0.18) | Intercept  Linear  Quadratic | -2.779  -0.040  0.002 | (0.168)  (0.034)  (0.001) | <0.001  0.236  0.037 |
| 2 “Poor at birth, non-poor at age 30” | 555 (15.9) | 0.60 (0.15) | Intercept  Linear  Quadratic  Cubic | -0.968  -0.171  0.019  -0.0004 | (0.296)  (0.085)  (0.007)  (0.0002) | 0.001  0.045  0.007  0.006 |
| 3 “Non-poor at birth, poor at age 30” | 487 (13.9) | 0.67 (0.15) | Intercept  Linear  Quadratic  Cubic | -0.069  0.415  -0.041  0.001 | (0.153)  (0.090)  (0.008)  (0.0002) | 0.651  <0.001  <0.001  <0.001 |
| 4 “Always poor” | 722 (20.6) | 0.74 (0.17) | Intercept  Linear  Quadratic | 1.003  0.056  -0.002 | (0.171)  (0.027)  (0.001) | <0.001  0.040  0.014 |

*Note*: sd = standard deviation; se = standard error

**Supplementary Figure S1.** Trajectories of family income by child´s age.
